# Reovirus FAST Proteins Drive Pore Formation and Syncytiogenesis Using a Novel Helix-Loop-Helix Fusion-Inducing Lipid Packing Sensor

**DOI:** 10.1371/journal.ppat.1004962

**Published:** 2015-06-10

**Authors:** Jolene Read, Eileen K. Clancy, Muzaddid Sarker, Roberto de Antueno, David N. Langelaan, Hiren B. Parmar, Kyungsoo Shin, Jan K. Rainey, Roy Duncan

**Affiliations:** 1 Department of Microbiology and Immunology, Dalhousie University, Halifax, Nova Scotia, Canada; 2 Department of Biochemistry and Molecular Biology, Dalhousie University, Halifax, Nova Scotia, Canada; 3 Department of Chemistry, Dalhousie University, Halifax, Nova Scotia, Canada; 4 Department of Pediatrics, Dalhousie University, Halifax, Nova Scotia, Canada; University of Kentucky, Lexington, UNITED STATES

## Abstract

Pore formation is the most energy-demanding step during virus-induced membrane fusion, where high curvature of the fusion pore rim increases the spacing between lipid headgroups, exposing the hydrophobic interior of the membrane to water. How protein fusogens breach this thermodynamic barrier to pore formation is unclear. We identified a novel fusion-inducing lipid packing sensor (FLiPS) in the cytosolic endodomain of the baboon reovirus p15 fusion-associated small transmembrane (FAST) protein that is essential for pore formation during cell-cell fusion and syncytiogenesis. NMR spectroscopy and mutational studies indicate the dependence of this FLiPS on a hydrophobic helix-loop-helix structure. Biochemical and biophysical assays reveal the p15 FLiPS preferentially partitions into membranes with high positive curvature, and this partitioning is impeded by bis-ANS, a small molecule that inserts into hydrophobic defects in membranes. Most notably, the p15 FLiPS can be functionally replaced by heterologous amphipathic lipid packing sensors (ALPS) but not by other membrane-interactive amphipathic helices. Furthermore, a previously unrecognized amphipathic helix in the cytosolic domain of the reptilian reovirus p14 FAST protein can functionally replace the p15 FLiPS, and is itself replaceable by a heterologous ALPS motif. Anchored near the cytoplasmic leaflet by the FAST protein transmembrane domain, the FLiPS is perfectly positioned to insert into hydrophobic defects that begin to appear in the highly curved rim of nascent fusion pores, thereby lowering the energy barrier to stable pore formation.

## Author Summary

The fusogenic ortho- and aquareoviruses are the only known nonenveloped viruses that induce syncytium formation. Cell-cell fusion is a virulence determinant of fusogenic reoviruses, and is mediated by a singular family of fusion-associated small transmembrane (FAST) proteins, the smallest known viral fusogens. Unlike their enveloped virus counterparts, reovirus FAST proteins have exceptionally small ectodomains and considerable larger cytoplasmic endodomains, suggesting FAST protein interactions with the cytoplasmic leaflet of the plasma membrane likely play a prominent role in the fusion process. We determined that the baboon reovirus p15 FAST protein endodomain contains a novel type of helix-loop-helix lipid packing sensor that partitions into hydrophobic defects present in highly curved membranes. This fusion-inducing lipid packing sensor (FLiPS) is required for pore formation, and can be functionally replaced by heterologous lipid packing sensors. By masking hydrophobic defects appearing in the highly curved rim of nascent fusion pores, the FliPS would make the forward reaction to pore formation a more energetically favored means of resolving an unstable hemifusion intermediate. These results define a new role for curvature sensing motifs, and reveal how viral fusion proteins can drive pore formation without having to rely on membrane stresses induced by complex refolding of large ectodomains.

## Introduction

The fusogenic ortho- and aquareoviruses are the only known examples of nonenveloped viruses that induce syncytium formation. Reovirus-induced syncytiogenesis is mediated by a novel family of viral fusogens, the fusion-associated small transmembrane (FAST) proteins [1, 2]. Structurally and functionally, FAST proteins differ dramatically from the three well-characterized classes of enveloped virus fusion proteins [3]. Ranging from ~100–200 residues in size, FAST proteins are considerably smaller than enveloped virus fusion proteins, and they position the majority of their mass within their single transmembrane domain and cytoplasmic tail rather than in their ectodomains [4–9]. FAST protein-mediated membrane fusion must therefore proceed in the absence of the complex ectodomain refolding used by enveloped virus fusogens to drive the fusion process [10]. Furthermore, unlike enveloped virus fusogens, FAST proteins are nonessential for reovirus replication, and as nonstructural viral proteins are not involved in virus entry. Instead, FAST proteins localize to the plasma membrane of virus-infected cells where they mediate cell-cell, not virus-cell, membrane fusion. The fusogenic reoviruses use syncytium formation to promote direct cell-cell transmission of the infection and rapid release of intracellular progeny virions following disruption of syncytia, both of which may contribute to the natural pathogenicity of the fusogenic reoviruses [11–13]. The unique structural and functional attributes of the FAST proteins and their link to virulence underscore the need for a better understanding of how these diminutive viral fusogens induce syncytium formation.

Studies of archetypal enveloped virus membrane fusion proteins converge on a common pathway of protein-mediated membrane fusion [14–16]. In this fusion-through-hemifusion pathway, small hydrophobic or amphipathic fusion peptides or fusion loops are proposed to shallowly insert into the outer leaflet of the membrane bilayer, forcing apart lipid headgroups and resulting in formation of a protruding membrane dimple [17, 18]. Homotypic fusion of endoplasmic reticulum membranes, synaptic vesicle exocytosis, and formation of clathrin-coated pits all rely on membrane insertion of amphipathic helices (AHs) or hydrophobic loops to generate membrane curvature via a similar wedging mechanism [19–22]. In the case of membrane fusion, the resulting membrane stresses and lipid packing defects are partially relieved by lipid mixing between outer leaflets of the closely apposed bilayers to generate a hemifusion stalk intermediate [23, 24], with curvature stresses in the stalk being relieved by subsequent pore formation [18]. Theoretical and experimental studies suggest that the highly curved rim of nascent fusion pores forces apart lipid headgroups, exposing the membrane interior to water, and that pore formation is the rate-limiting step in the fusion process [25–28]. At present, there is no clear understanding of how protein fusogens breach this barrier.

Members of the FAST protein family have limited sequence similarity, but share a number of defining features. A single transmembrane domain separates exceedingly small N-terminal ectodomains of ~19–40 residues from equal sized or larger C-terminal cytoplasmic endodomains of ~40–140 residues [1]. Each FAST protein contains a cytoplasmic cluster of membrane-proximal basic residues, which in the case of the p14 FAST protein function as a Golgi export signal, [29], and all contain cites for acylation; either myristoylation of the N-terminal Gly residue or palmitoylation of endodomain Cys residues [4, 30, 31]. All three FAST protein domains (ecto-, endo- and transmembrane) are actively involved in the fusion process and are somewhat interchangeable between family members [32–34], indicating the FAST proteins evolved as modular fusogens. How each fusion module promotes membrane fusion is still unclear, but several essential structural motifs have been characterized. For example, ectodomains of the avian reovirus p10, reptilian reovirus p14 and baboon reovirus p15 FAST proteins contain a cystine loop, proline-hinged loop, or polyproline type II helix, respectively [35–38]. All three of these motifs are structurally distinct from, but functionally equivalent to, enveloped virus fusion peptides [3], using membrane insertion of hydrophobic residues to promote liposome fusion. While the fusion peptides of enveloped viruses and FAST proteins are clearly involved in inducing membrane curvature needed for hemifusion, how protein fusogens promote curvature changes required for pore formation has not been established.

A remarkable feature of FAST proteins is their asymmetric membrane topology, which positions the majority of their mass on the distal side of the contacting membrane bilayers [1]. These cytoplasmic tails play diverse roles in syncytiogenesis. Truncation and substitution studies revealed the membrane-distal region of the endodomain is required for pore formation and augments pore expansion [32]. The sequence-independence of the distal portion of the endodomain suggests this region may function as an intrinsically disordered motif, a common property of motifs with multiple interaction partners [39]. A recent study identified intracellular annexin A1 as one such interaction partner of the p14 FAST protein; annexin A1 functions in a calcium-specific manner to promote pore expansion [40]. Following proteolytic cleavage, the p14 FAST protein endodomain can also function in a sequence-specific fashion in *trans* to augment pore expansion following cell-cell fusion mediated by the full-length p14 protein [41]. As with annexin A1, the soluble p14 endodomain enhances pore expansion following membrane fusion induced by fusogens other than just FAST proteins [40, 41], suggesting syncytium formation may be a generalized cellular response to resolve the unfavorable architecture of intercellular pores.

We now report a new functional motif in the FAST protein cytoplasmic tails that is required for pore formation. In addition to their transmembrane domain, all FAST proteins contain a small cluster of predominantly hydrophobic or apolar residues, termed the hydrophobic patch (HP). While the HP is contained in the ectodomains of the p10 and p14 FAST proteins where it functions as a fusion peptide [35, 36, 42, 43], this motif is located in the endodomains of other FAST proteins and has no known function. Results indicate the membrane-proximal, endodomain HP of the p15 FAST protein (p15 HP) functions as a lipid packing sensor and preferentially partitions into highly curved membranes. By promoting or stabilizing such curvature in the inner leaflet of the plasma membrane, this curvature sensor provides a mechanism to lower the energy barrier to pore formation and promote cell-cell fusion.

## Results

### The cytosolic p15 HP is essential for syncytium formation

**Fig 1:**
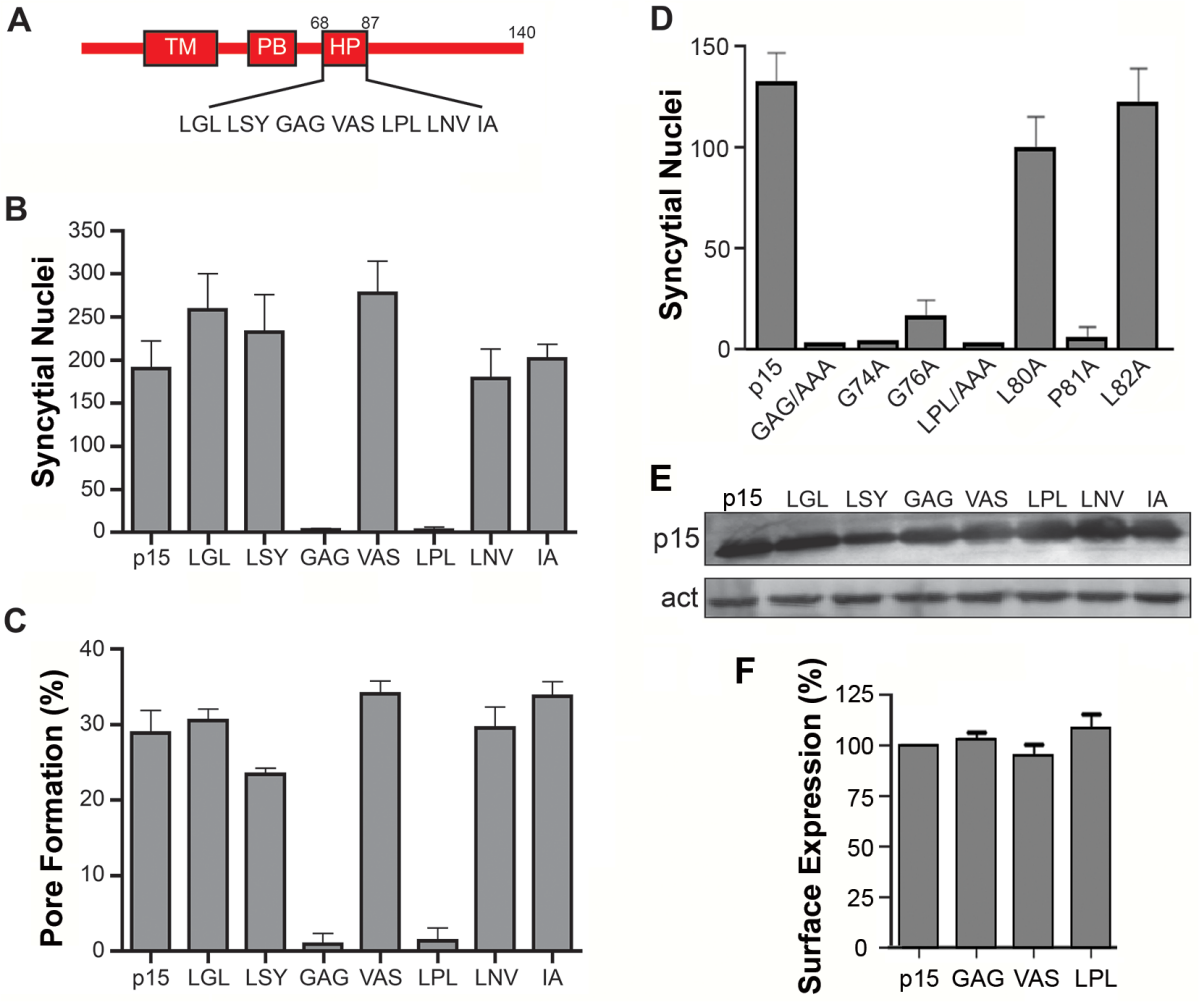
Centrally-located glycine and proline residues in the p15 HP are essential for cell-cell fusion. (A) Schematic of the p15 protein indicating locations of the transmembrane domain (TM), polybasic cluster (PB) and hydrophobic patch (HP). Numbers indicate residue positions in p15. Sequence of the HP is depicted below. (B) Transfected QM5 cells expressing wt p15 or p15 mutant proteins containing triple Ala substitutions of the indicated three consecutive HP residues were Giemsa-stained at 9 h post-transfection and the mean ± SEM of syncytial nuclei per microscopic field were quantified by bright field microscopy. (C) QM5 cells co-transfected with EGFP and wt p15 or the indicated p15 HP mutant proteins as described in panel B were co-cultured with non-transfected cells labeled with calcein red, then sorted by flow cytometry to quantify the percentage of dual fluorescent cells indicative of pore formation. Results are the means ± SEM of Overton subtractions using duplicate samples from n = 2 independent experiments. (D) QM5 cells expressing wt p15 (p15) or p15 mutant proteins containing Ala substitutions of the indicated HP residues constructs were processed as in panel B to quantify syncytium formation. (E) Western blot of QM5 cell lysates expressing wt p15 or p15 mutant proteins containing triple Ala substitutions of the indicated HP residues HP were probed with anti-p15 antiserum and anti-actin. (F) Cell surface fluorescence of QM5 cells expressing wt p15 (p15) or p15 mutant proteins containing Ala substitutions of the indicated HP residues was quantified by flow cytometry using anti-p15 antiserum and Alexa Fluor 647-conjugated secondary antibody. Results are percent surface fluorescence relative to wt p15. Bar graphs in panels B, D and E are the mean ± SEM for triplicate samples from n = 3 experiments.

The majority of FAST proteins contain a membrane-proximal HP in their cytoplasmic endodomains [2]. The function of these motifs has not been investigated. To discern whether the p15 HP plays any role in syncytium formation, HP residues were replaced by Ala in groups of three consecutive amino acids. Syncytium formation in transfected QM5 cells expressing individual p15 HP mutant proteins was quantified using a standard syncytial assay based on the average number of syncytial nuclei/microscopic field [4]. Ala substitution of two regions in the p15 HP, GAG_74-76_ and LPL_80-82_, abrogated syncytium formation (Fig 1B). The non-syncytiogenic p15GAG_74-76_ and p15LPL_80-82_ constructs were also defective for pore formation (Fig 1C), as determined using a standard dual fluorescence assay based on flow cytometry to quantify transfer of EGFP and calcein red between donor and target cells [32]. To more precisely identify which residue(s) in the GAG_74-76_ and LPL_80-81_ regions of the p15 HP are essential for fusion, each residue was individually substituted with Ala. Substitutions of L80 or L82 had little to no effect on syncytiogenesis, while substitutions of P81, G74 or G76 essentially eliminated syncytium formation (Fig 1D). As shown by western blotting (Fig 1E) and flow cytometry analysis of cell surface localization, analyzed by percent cell fluorescence relative to wt p15 (Fig 1F) or by mean fluorescence intensity (S1 Fig), none of the triple Ala substitutions had any effect on p15 expression or trafficking to the plasma membrane. The p15 HP is therefore essential for cell-cell pore formation, and this activity is dependent on Gly and Pro residues near the center of the HP.

### Helical properties and hydrophobicity influence the function of the p15 HP

**Fig 2:**
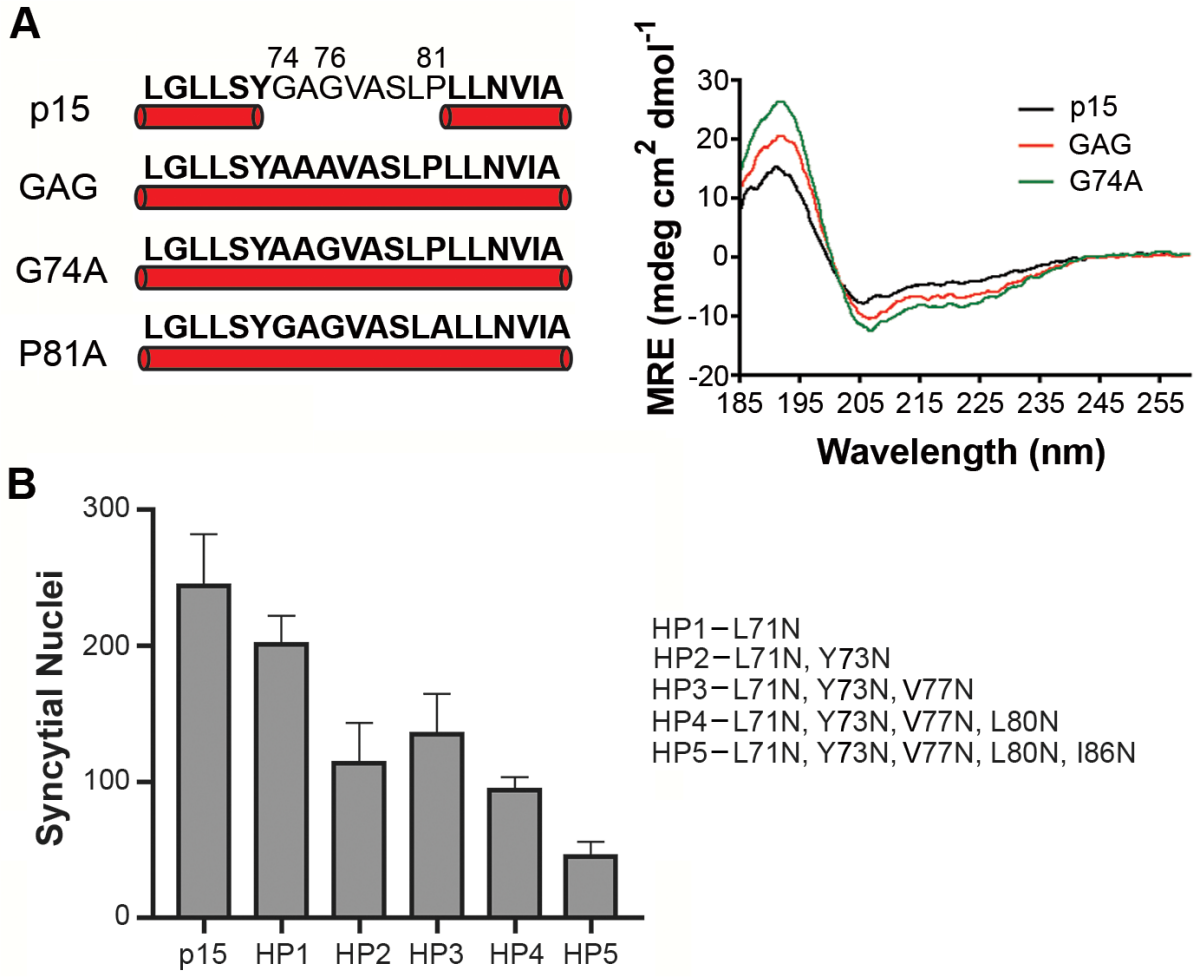
Helical properties and hydrophobicity influence the function of the p15 HP. (A) Secondary structure predictions (left panel) of the wt p15 HP sequence and the indicated p15 HP Ala substitutions (GAG contains a double substitution of G74/76A) as predicted using Phyre^2^ analysis (http://www.sbg.bio.ic.ac.uk/phyre2) of the complete p15 endodomain. Residues predicted to be -helical are bolded and underscored with a cylinder. Far-ultraviolet CD spectra (right panel) of wt p15HPpep or the Ala-substituted GAG or G74A p15HPpep peptides depicted on the left were obtained at 37°C in the presence of LPPG micelles. Measurements were collected from three runs in each of two independent experiments and converted to mean residue ellipticity (MRE). (B) QM5 cells expressing wt p15 protein or mutant p15 proteins (HP1-5) containing asparagine substitutions of HP residues indicated on the right were fixed at 10 h post transfection, Giemsa-stained, and quantified for the average number of syncytial nuclei. Results are mean SEM of triplicate samples from n = 3 independent experiments.

Secondary structure predictions [44] suggested a helix-loop-helix (H-L-H) conformation for the wild type (wt) p15 HP, while the fusion-dead p15GAG_74-76_, G74A and p15P_81_A constructs were predicted to be a continuous α-helix (Fig 2A). These predictions were experimentally confirmed using circular dichroism (CD) spectroscopy of synthetic peptides corresponding to the wt p15HP (p15HPpep) and G74/76A (GAG) and G74A mutant versions of this peptide. CD spectra revealed both mutant peptides assumed more helical structure in the presence of 1-palmitoyl-2-hydroxy-sn-glycero-3-[phospho-rac-(1-glycerol)] (LPPG) micelles, as evidenced by increased maxima at 190 nm and increased minima at 208 nm and 222 nm relative to the wt peptide (Fig 2A). These shifts in the CD spectra of the peptides imply increased helicity and suggest conversion of at least part of the loop region to an α-helix, with the Pro81 residue likely preventing complete helix formation. Several attempts to synthesize and purify a peptide containing the P81A substitution were unsuccessful.

As its name suggests, the p15 HP comprises mostly hydrophobic or apolar residues (16 of 20 residues). The functional significance of hydrophobic residues in the p15 HP was supported by substitution studies in the context of the p15 protein, sequentially replacing residues in the predicted N- and C-terminal helices with the uncharged, polar residue asparagine. Asparagine replacement of the N-terminal L71 residue reduced p15-induced syncytium formation by ~20%, and when combined with a similar replacement of Y73 in the predicted N-terminal helix by ~50% (constructs HP1 and HP2 in Fig 2B). Additional replacements of V77 and L80 in the predicted loop region had no further inhibitory effect (constructs HP3 and HP4), but additional substitution of I86 in the predicted C-terminal helix reduced syncytium formation by ~80% (construct HP5 Fig 2B). These results suggest that changes in the predicted helicity and hydrophobicity of the p15 HP compromise its role in cell-cell fusion.

### Helix-loop-helix conformation of p15HPpep in a membrane mimetic environment

The functional implications of a predicted H-L-H p15 HP architecture prompted determination of the atomic-level structure of a 20-residue p15HPpep synthetic peptide (L_68_GLLSYGAGVASLPLLNVIA_87_; numbers refer to the residue location in p15) using solution-state nuclear magnetic resonance (NMR) spectroscopy. High-resolution NMR spectra of p15HPpep implied a uniform, homogeneous structure in dodecylphosphocholine (DPC) micelles, as indicated by a single observed set of resonance frequencies for each spin system (S2 Fig). The ^1^H-^1^H pairwise distance restraints used in the structure calculation were generated from observed Nuclear Overhauser Effects (NOEs) (S1 Table). Approximately 99% of the expected ^1^H chemical shifts were determinable using sequential assignment protocols [45] based upon 2D ^1^H-^1^H TOCSY and 2D ^1^H-^1^H NOESY experiments (S2A Fig). Natural abundance 2D ^1^H-^15^N and 2D ^1^H-^13^C HSQC spectra allowed for complete amide ^15^N and α/β ^13^C chemical shift determination (S2B and S2C Fig). Following an iterative NOE distance restraint refinement protocol [46], the final structural ensemble of p15HPpep was calculated using 511 NOE-based distance restraints (S1 Table). The 50 lowest-energy structures (out of 100 calculated) were retained, showing excellent agreement with NOE data and reasonable Ramachandran plot statistics (S1 Table).

**Fig 3:**
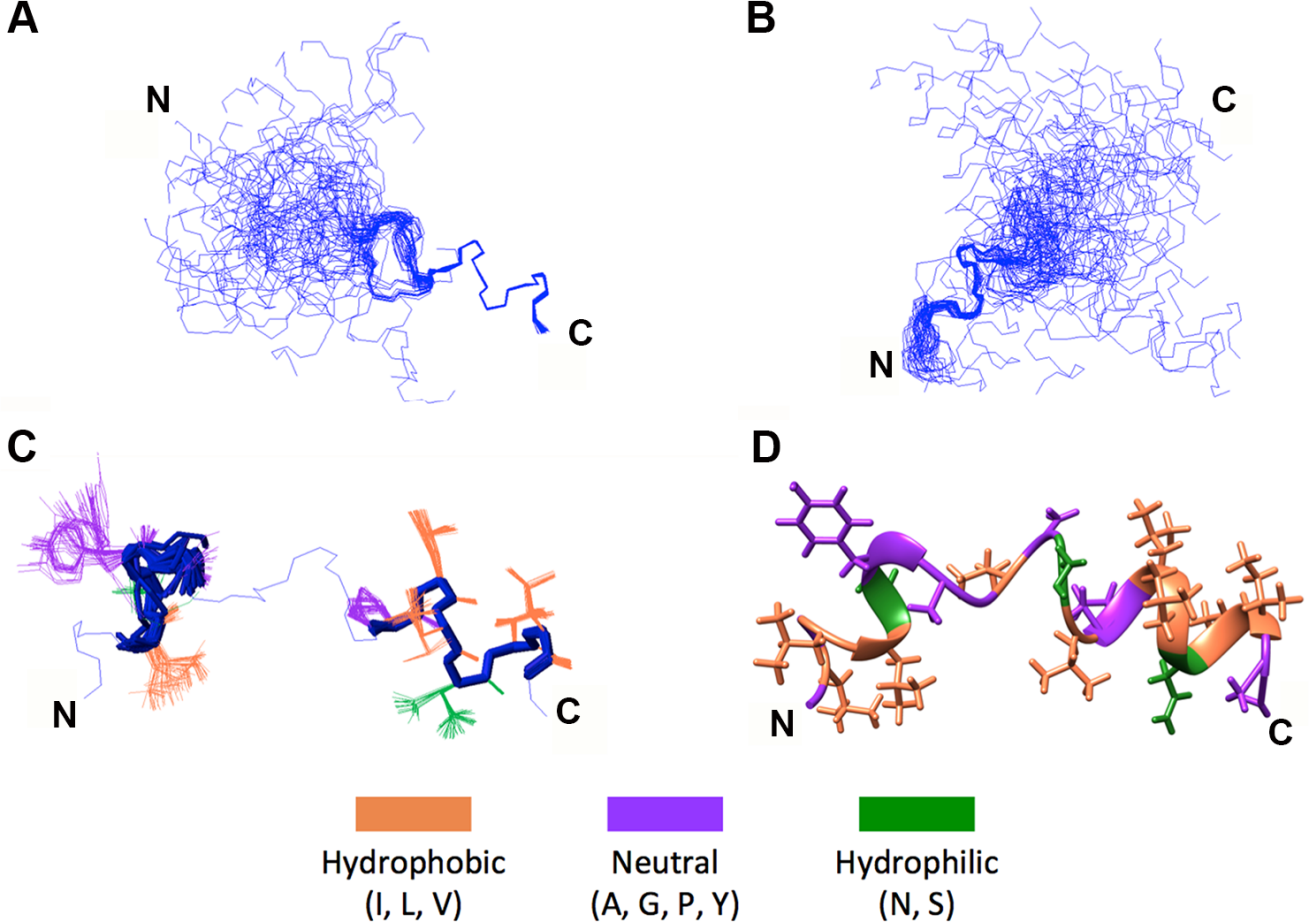
NMR structural determination of a helix-loop-helix conformation in p15HPpep. (A) Ensemble of the 50 lowest energy structures (out of 100 calculated) of wt p15HPpep with the C-terminal P81-I86 helix superposed. (B) Ensemble of the 50 lowest energy structures with the N-terminal L70-G74 helix superposed. (C) Backbone superposition of the two terminal p15HPpep helices (L70-G74 and P81-I86) of all 50 ensemble members onto the lowest-energy conformer. (D) Lowest energy conformer of the wt p15HPpep. In this conformer, the α-helical segments span residues L71-G74 and P81-I86 at the termini, connected by an unstructured turn/loop. Residues in panels C and D are color-coded as indicated.

The 50-member solution NMR structural ensemble indicated almost all of the p15HPpep conformers contain two short α-helical segments at the N- and C-termini connected by an unstructured turn/loop (Fig 3C). Due to the turn/loop between helices and lack of long-range distance restraints between the helices, the orientation of the two helices relative to each other was not constrained, as clearly evident when conformers where arranged by overlaying the N- or C-terminal helices (Fig 3A and 3B). The C-terminal helix spanning residues L82-I86 was consistent throughout the ensemble; this helix extended to P81 in 38 of the 50 members. Positioning of L82, L83, V85 and I86 side chains on the same face of this C-terminal helix forms a hydrophobic face (Fig 3D). The N-terminal helix exhibited a higher degree of variability throughout the ensemble (Fig 3C), but spanned residues L71-Y73 in 37 of the 50 members; in 33 of these 37 members the helix extended to include L70 and/or G74. Notably, no ensemble member contained a continuous helix spanning residues G74, G76 and P81, the three residues where Ala substitutions in the context of the p15 protein eliminated membrane fusion (Fig 1D). Structural and functional studies therefore indicate a H-L-H conformation is a functional requirement of the p15 HP.

### The p15HPpep peptide preferentially partitions into highly curved lipid bilayers containing hydrophobic defects

**Fig 4:**
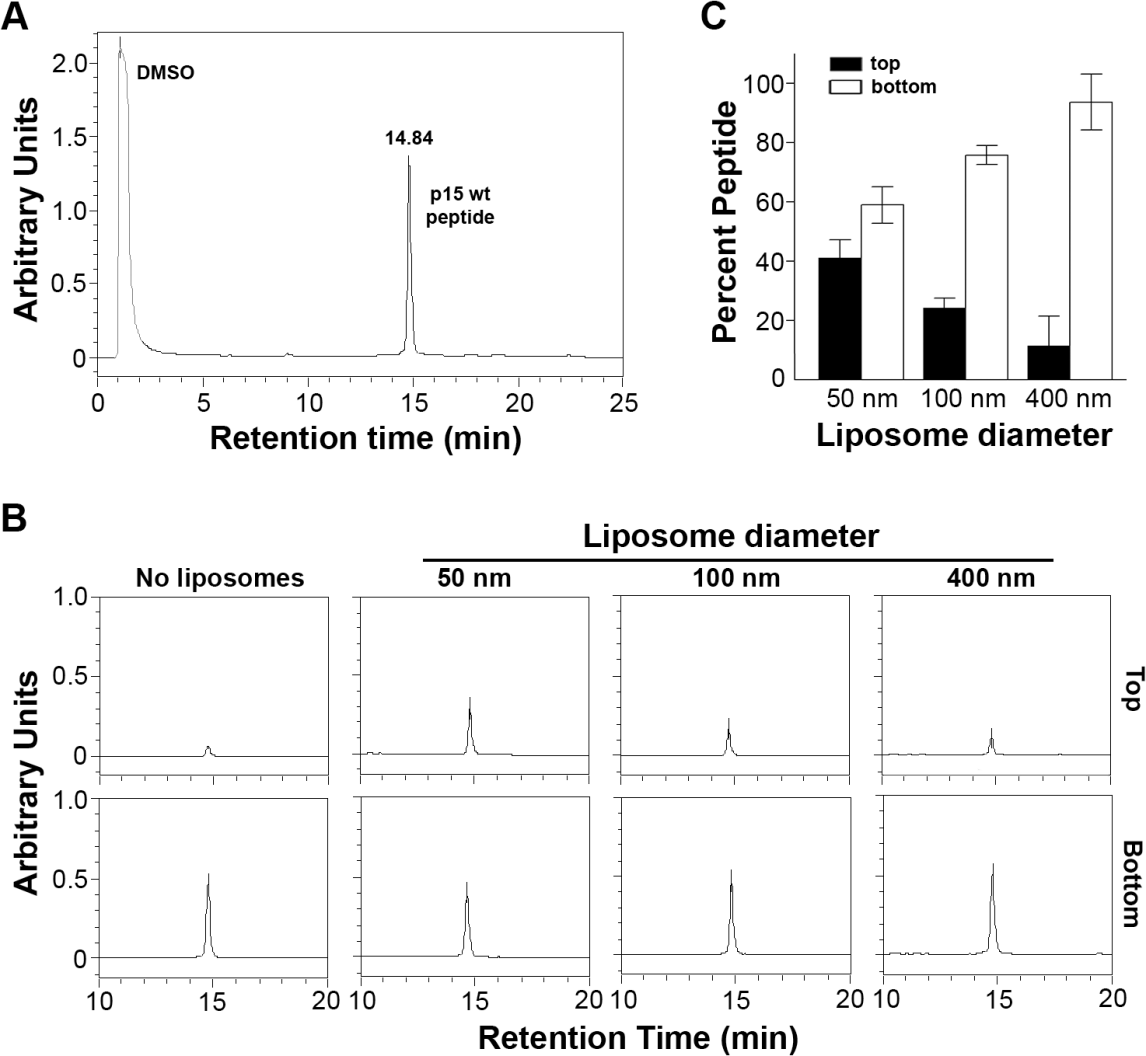
The p15HPpep peptide preferentially partitions into highly curved membranes. (A) Purified wt p15HPpep dissolved in DMSO was resolved by reverse-phase HPLC using a water:acetonitrile gradient and eluted with the indicated retention time. The chromatogram presents the area under the peaks in arbitrary units of absorbance at 215 nm. (B) The wt p15HPpep was mixed with liposomes (1:1:1 DOPC:DOPE:cholesterol) of the indicated diameters at a fixed peptide:lipid molar ratio (1:500), and liposomes were separated from free peptide by flotation on sucrose gradients. The peptides present in the top liposome and bottom free peptide fractions were detected by HPLC. Chromatograms from a representative experiment are shown in arbitrary fluorescence units at the same scale for the retention time corresponding to the p15HPpep. The No Liposome chromatograms are for p15HPpep treated exactly as above but incubated in the absence of liposomes before sucrose gradient fractionation; the chromatogram indicates the low level of free peptide contaminating the top sucrose fraction. (C) The liposome (top) and free peptide (bottom) sucrose fractions were analyzed by HPLC as in panel B, and the relative peptide concentration in each fraction quantified by integrating the area under the peak in the HPLC chromatograms. Percent peptide = Area_Top_ / Total Area_Top+Bottom_. Bars represent the mean SEM of three experiments.

To examine the membrane interactive properties of the p15 HP, wt p15HPpep was incubated with liposomes of varying diameters (50, 100 or 400 nm) at a constant peptide:lipid molar ratio (1:500). Liposomes were separated from free peptide by flotation using sucrose density gradient ultracentrifugation, and reverse phase HPLC was used to quantify peptide concentrations in the top 0–20% sucrose interface and bottom 30% sucrose gradient fractions, corresponding to liposome-associated and free peptide, respectively. Control experiments confirmed different sized liposomes were all recovered at 90–95% efficiency from the 0–20% sucrose interface (S3A Fig), and HPLC analysis of different peptide concentrations defined a linear dose-response range for peptide quantification (S3B Fig). Results indicated a progressive, size-dependent increase in p15HPpep association with liposomes at the 0–20% sucrose interface (Fig 4B). Replicate experiments (n = 3) indicated ~5% of total p15HPpep associated with 400 nm liposomes, ~20% with 100 nm liposomes, and ~40% with 50 nm liposomes (Fig 4C). When corrected for background peptide present in 0–20% sucrose interface in the absence of liposomes (~3% of total peptide, Fig 4B), decreasing liposome size from 400 nm to 50 nm resulted in an ~4-fold increase in p15HPpep partitioning into liposomes. Thus, the p15HPpep peptide preferentially partitions into lipid bilayers with a high degree of positive curvature.

**Fig 5:**
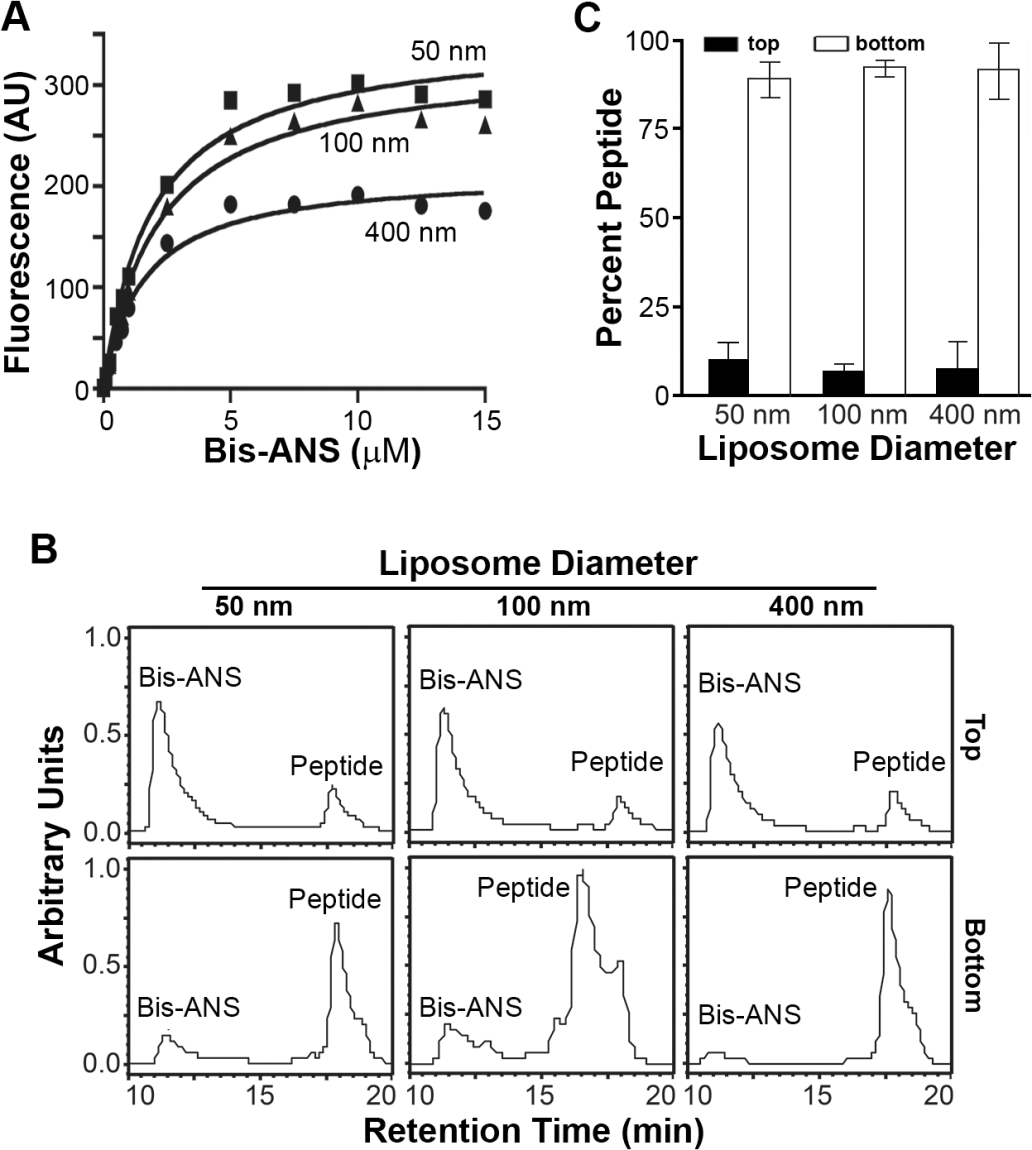
Partitioning of p15HPpep into liposomes is based on curvature-dependent hydrophobic defects. (A) Partitioning curves of bis-ANS in 100 mM suspensions of 50 nm, 100 nm, and 400 nm liposomes. bis-ANS fluorescence was fit to one-site binding hyperbola nonlinear regression using GraphPad Prism. Data points represent the mean of three experiments, with error bars contained within the symbols on the graph. (B) HPLC chromatograms in arbitrary units of absorbance at 215 nm of the top (liposomes) and bottom (free peptide) fractions from sucrose gradients following partitioning of wt p15HPpep into liposomes of the indicated diameter as described in Fig 4A, except liposomes were pre-treated with 10 mM bis-ANS before p15HPpep addition. Note that retention times of the wt p15HPpep differ from Fig 4 due to use of a different HPLC column. (C) As in panel B, quantifying percent p15HPpep associated with top (black bars) and bottom (white bars) sucrose fractions based on integrating the area under the chromatogram peaks. Results are mean SEM from three independent experiments.

To determine whether the p15 HP might function as a “curvature sensor” to detect hydrophobic defects in highly curved lipid bilayers, the small molecule fluorescent hydrophobic probe bis-ANS (4,4'-dianilino-1,1'-binaphthyl-5,5'-disulfonic acid) was used as a partitioning competitor. Analysis of bis-ANS partitioning into liposomes (bis-ANS fluorescence increases in non-polar environments) indicated increased bis-ANS partitioning maxima as liposome size decreased from 400 nm to 50 nm (Fig 5A). These results confirmed small liposomes (<100 nm diameter) have more hydrophobic defects than larger liposomes, and they determined 10 μM bis-ANS saturates hydrophobic defects in the different sized liposomes. Pre-treatment of liposome suspensions with 10 μM bis-ANS before peptide addition essentially eliminated the size-dependent partitioning of the p15HPpep peptide (Fig 5B and 5C); p15HPpep partitioned into liposomes at levels only slightly above background (~5–10% of total peptide) and independent of liposome size. The p15HPpep peptide therefore senses membrane curvature via hydrophobic defects in highly curved lipid bilayers.

**Fig 6:**
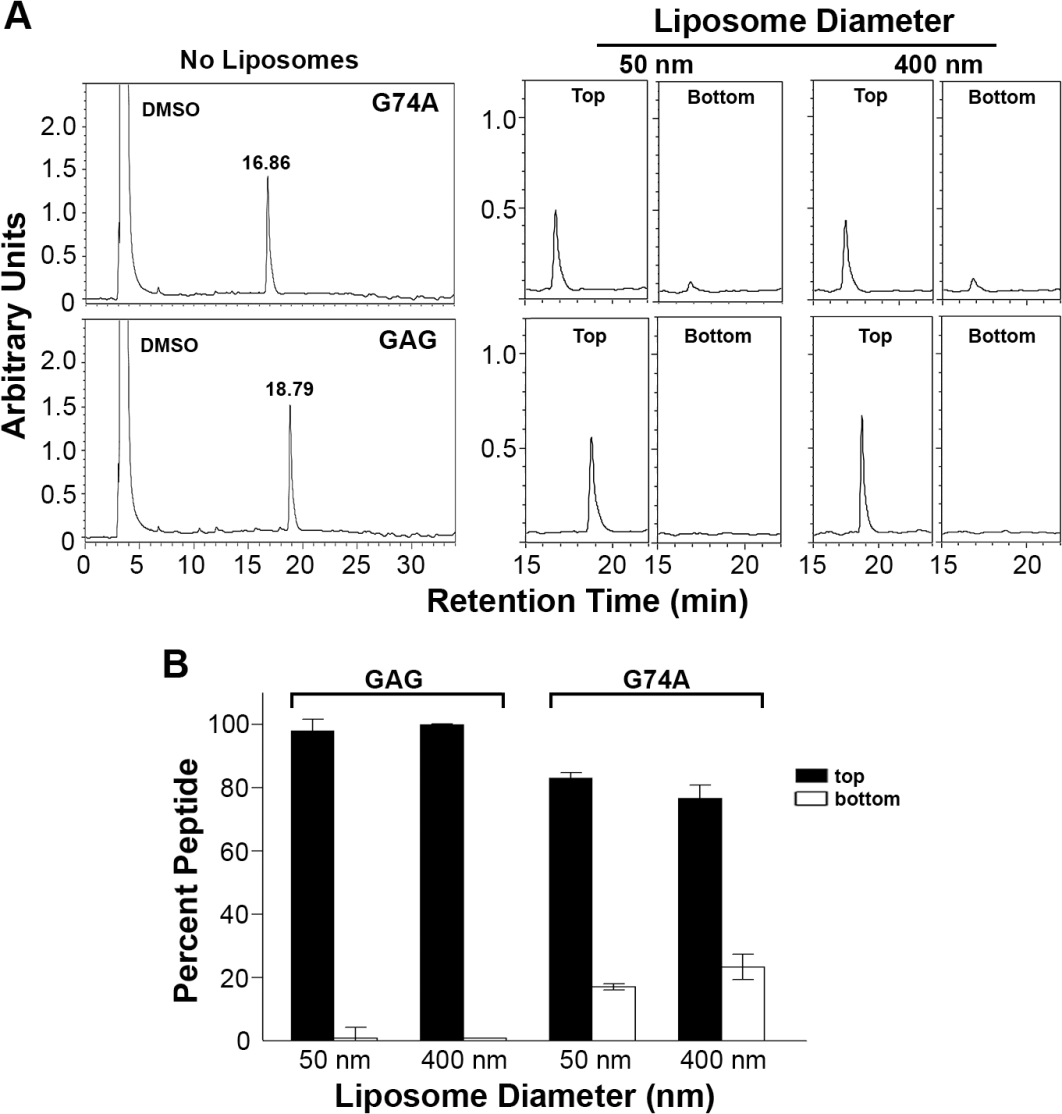
Mutant p15HPpep containing substitutions that disrupt the helix-loop-helix conformation partition into liposome membranes indiscriminate of membrane curvature. (A) Purified mutant p15HPpep peptides (G74A and G74/76A [GAG]) dissolved in DMSO were resolved by reverse-phase HPLC using a water:acetonitrile gradient and eluted with the indicated retention times (left panel). Peptides were mixed with liposomes (1:1:1 DOPC:DOPE:cholesterol) of the indicated diameters at a fixed peptide:lipid molar ratio (1:500) and liposomes were separated from free peptide by flotation on sucrose gradients. Peptides present in the top liposome and bottom free peptide fractions were detected by HPLC (right panel). Chromatograms from a representative experiment are shown in arbitrary fluorescence units at the same scale. (B) The liposome (top) and free peptide (bottom) sucrose fractions were analyzed by HPLC as in panel A, and the relative peptide concentration in each fraction quantified by integrating the area under the peak in the HPLC chromatograms. Percent peptide = Area_Top_ / Total Area_Top+Bottom_. Bars represent the mean SEM of three experiments.

To examine whether the H-L-H conformation of the p15 HP required for p15-mediated cell-cell fusion activity is also required for membrane curvature sensing, we used the two mutant p15HPpep peptides containing the G74/76A substitutions in the GAG sequence or just the G74A substitution. Both of these substitutions abrogated cell-cell fusion activity in the context of the p15 protein (Fig 1D) and increased helicity of the respective mutant p15HPpep peptides (Fig 2A). When examined using the liposome partitioning assay, both mutant peptides lost their ability to function as a membrane curvature sensor. In marked contrast to wt p15HPpep, in replicate experiments (n = 3) the mutant peptides avidly and equally associated with both 50 nm and 400 nm liposomes. Essentially all (>95%) of the GAG mutant peptide associated with both sizes of liposomes; ~80% of the G74A peptide showed similar size-indiscriminate association with liposomes (Fig 6). Hence, loss of the p15HPpep HLH conformation correlates with both a loss of syncytium formation induced by the p15 protein and loss of membrane curvature sensing by p15HPpep peptides.

### Heterologous lipid-packing sensors can functionally replace the p15 HP

**Fig 7:**
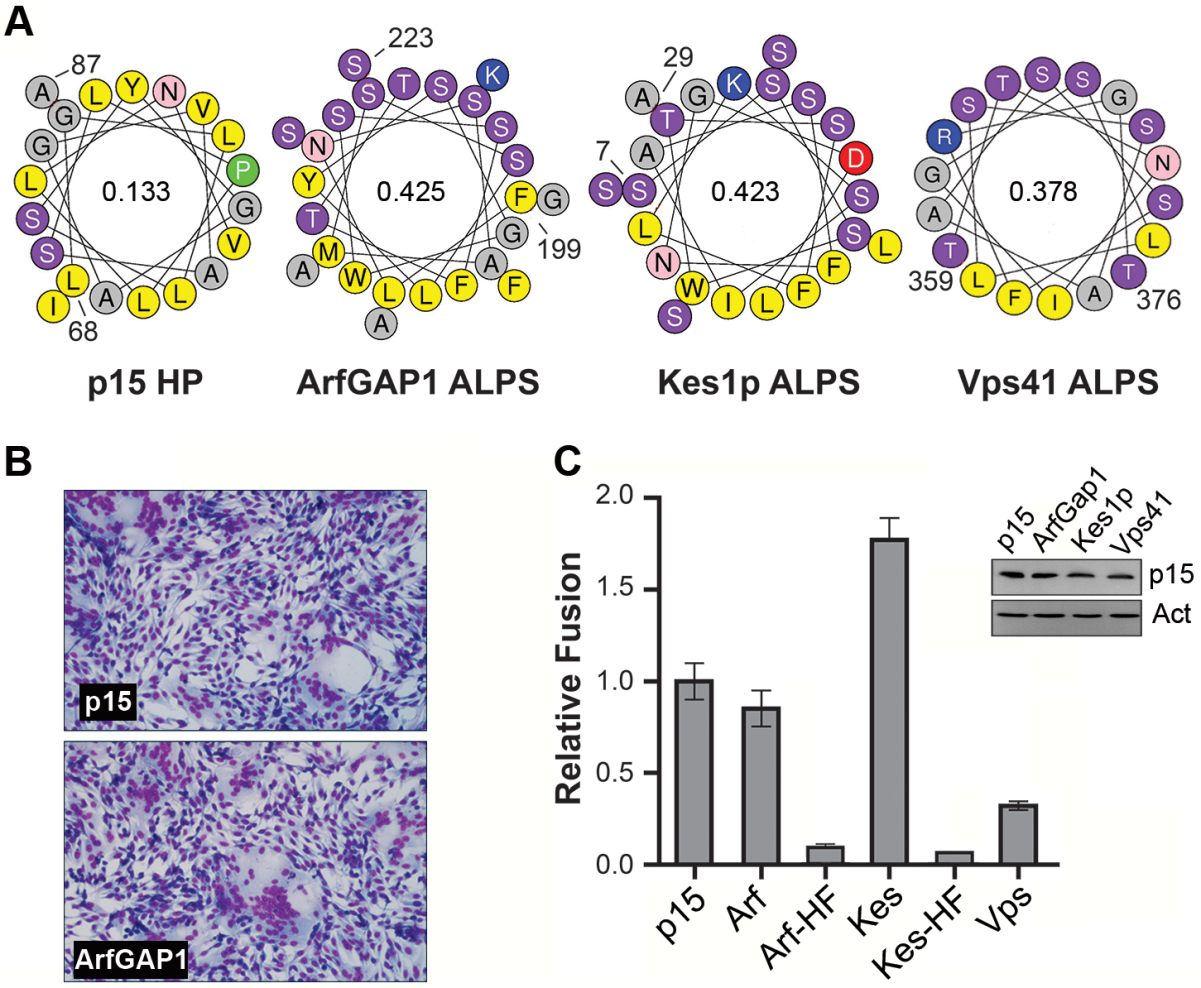
ALPS motifs can functionally replace the p15 HP. (A) Helical wheel representations of the p15 HP and ALPS motifs from the indicated proteins. Numbers indicate the location of these motifs in their respective proteins. Color code: yellow, hydrophobic; purple, serine and threonine residues; grey, Gly and Ala residues; blue, basic residues; red, acidic residues; pink, asparagine; green, proline. Numbers inside the helical wheels indicate the mean hydrophobic moment calculated using HeliQuest (http://heliquest.ipmc.cnrs.fr/). (B) Giemsa-stained micrographs of QM5 cells expressing wt p15 protein or chimeric p15 containing the ArfGap1 ALPS replacing the p15 HP taken at 10 h post-transfection. (C) Relative fusogenicity of p15 proteins containing replacement of the p15 HP with the ALPS motifs indicated in panel A. Arf-HF and Kes-HF are ALPS motifs containing Ala substitutions of three residues in the hydrophibic face (L200, W211, and F221 in ArfGAP1; W10, F13, and F20 in Kes1p). Results from a representative experiment (n = 2) are presented as the levels of syncytium formation SD relative to wt p15 based on quantifying syncytial nuclei in triplicate samples. The inset shows a western blot of equivalent protein loads of lysates from cells expressing the indicated p15 constructs probed with anti-p15 or -actin.

Well-characterized amphipathic lipid packing sensors (ALPS) respond to membrane curvature by partitioning linear AHs into lipid packing defects present in highly curved membranes [47–49]. The p15 HP is the first example of lipid packing sensor requiring a H-L-H conformation. Calculations of the mean hydrophobic moment, an indication of the relative amphipathic nature of a helix [50], also indicates the p15 HP motif is considerably less amphipathic than classical ALPS motifs (Fig 7A). Nonetheless, the p15HPpep peptide shares the curvature sensing capability of these heterologous ALPS motif peptides. To determine whether ALPS motifs can functionally replace the p15 HP during cell-cell membrane fusion, a series of chimeric p15 constructs containing various ALPS motifs in place of the p15 HP were created and assessed for their syncytiogenic activity. These ALPS motifs were from the membrane curvature sensing modules of Golgi-localized ArfGAP1 (F_199_LNSAMSSLYSGWSSFTTGASKFAS_223_), the sterol transporter protein Kes1p (S_7_SSWTSFLKSIASFNGDLSSLSA_29_), and the vacuole protein sorting effector subunit Vps41 (T_359_TNIGSLLSSAASSFRGT_376_) (Fig 7A). Most interestingly, all three ALPS motifs could functionally substitute for the p15 HP to varying degrees, independent of their relative expression levels (Fig 7B and 7C); ArfGAP1 yielded equivalent levels of syncytium formation as wt p15, Kes1p augmented p15 fusion activity, while p15 with the Vps41 motif retained modest, but significant, levels of cell-cell fusion activity. We note that Vps41 had the lowest hydrophobic moment and smallest hydrophobic face of the three ALPS motifs, although the relative fusion levels should be interpreted with caution in the absence of quantitative cell surface expression data and kinetic analysis of syncytium formation. Substitution of residues in the hydrophobic face of ALPS motifs that renders them insensitive to membrane curvature, L200A, W211A, and F221A in the ArfGAP1 motif and W10A, F13A, and F20A in the Kes1p motif [47, 51, 52], reduced syncytium formation of these p15 constructs by >95% (Fig 7C). The loss of cell-cell fusion activity was unlikely to be due to dramatic changes in protein expression or trafficking since three unrelated, heterologous ALPS motif can functionally replace the p15 HP, indicating the p15 HP is not required for p15 expression or trafficking to the plasma membrane. ALPS motifs that detect positive membrane curvature dependent on their hydrophobic face can therefore functionally substitute for the p15 HP.

**Fig 8:**
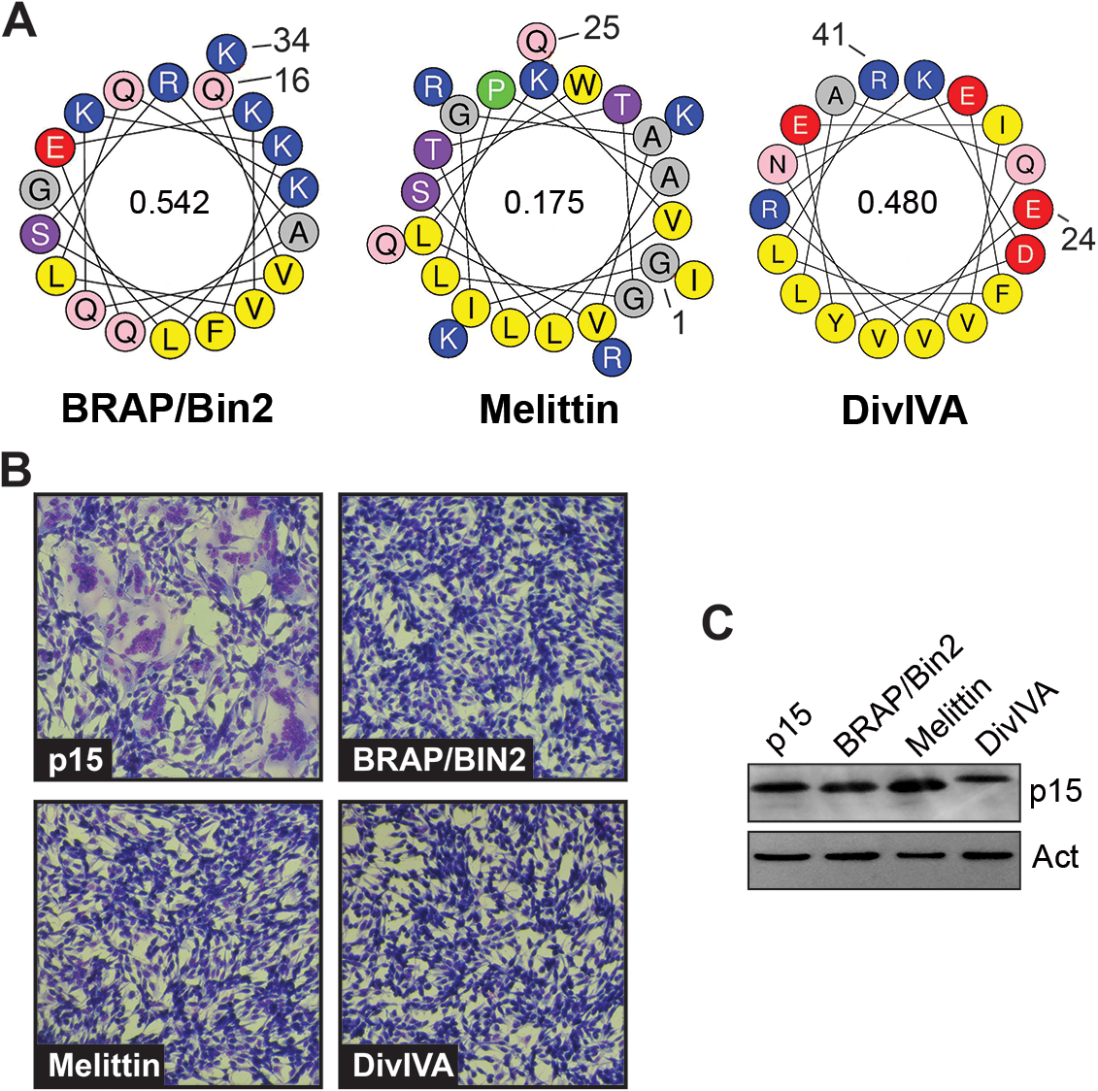
The p15 HP cannot be replaced with heterologous membrane-interactive amphipathic helices. (A) Helical wheel projections of the indicated amphipathic helices, color coded as in Fig 7. Numbers indicate the location of these motifs in their respective proteins, and numbers inside the helical wheels indicate the mean hydrophobic moment calculated using HeliQuest (http://heliquest.ipmc.cnrs.fr/). (B) Syncytium formation in Giemsa-stained, transfected QM5 cell monolayers expressing wt p15 or chimeric p15 constructs where the p15 HP was replaced by the indicated amphipathic helices. Images were acquired at 10 h post-transfection. (C) Western blot of equivalent protein loads of lysates from cells expressing the indicated p15 constructs probed with anti-p15 or anti-actin.

To ascertain if ALPS motifs are unique in their ability to functionally replace the p15 HP, three distinct membrane-interactive AHs were used to replace the p15 HP (Fig 8A). The H0-NBAR motif (Q_16_VQKKFSRAQEKVLQKLGK_34_) from BRAP/BIN2 has a high hydrophobic moment and strong positively charged face (Fig 8), but only partitions into membranes with negatively-charged lipid headgroups, independent of membrane curvature, and induces membrane rigidity [53]. The AH from melittin [G_1_IGAVLKVLTTGLPALSWI KRKRQQ_25_] is a lytic peptide that forms pores [54], and has a low hydrophobic moment (Fig 8). The AH of the *Bacillus subtilis* DivIVA protein (E_21_VNEFLAQVRKDYEIVLR_41_) has a high hydrophobic moment, but is involved in localizing DivIVA to negatively curved membranes [55]. Despite being expressed at levels similar to wt p15 (Fig 8C), none of these constructs were functional for syncytium formation (Fig 8B).

### A membrane-proximal amphipathic helix in the p14 FAST protein cytoplasmic tail serves a similar function as the p15 HP

**Fig 9:**
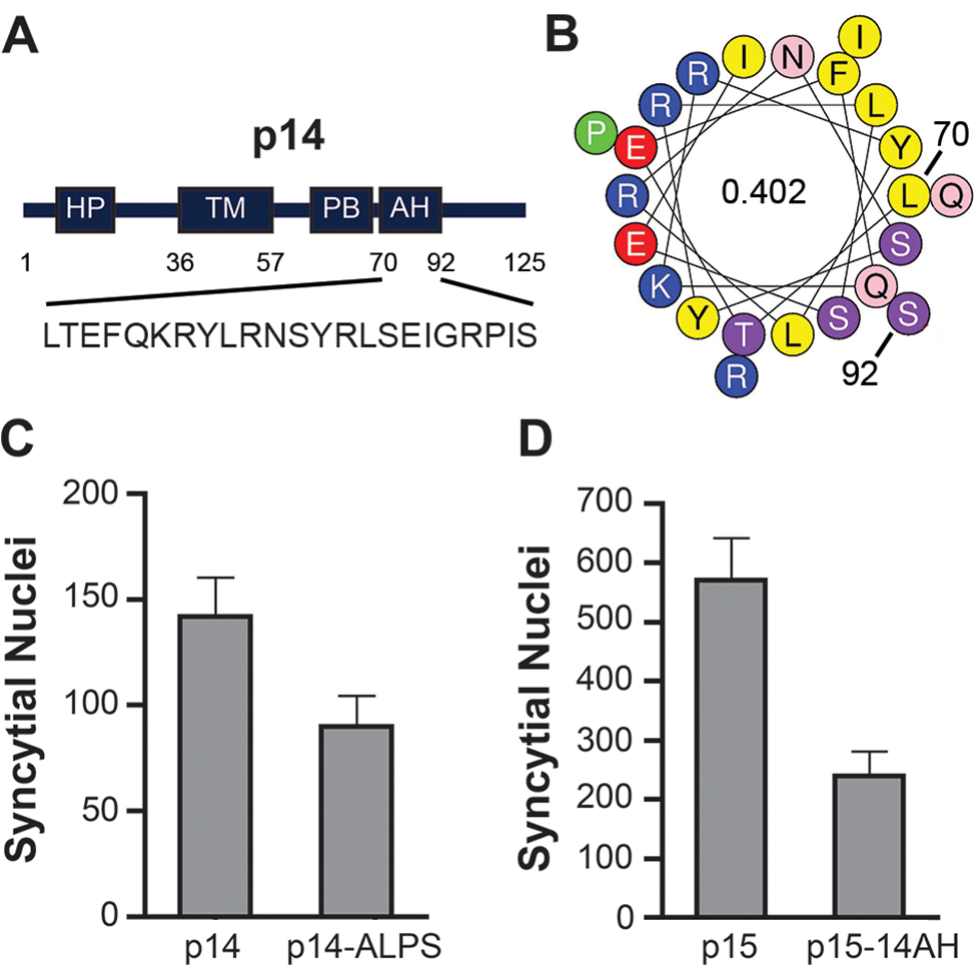
ALPS motif can functionally replace an amphipathic helix in the p14 FAST protein. (A) Schematic of the p14 FAST protein indicating the location of the ectodomain hydrophobic patch (HP), transmembrane domain (TM), polybasic cluster (PB), and amphipathic helix (AH). Numbers indicate residue position in p14, and numbers inside the helical wheels indicate the mean hydrophobic moment calculated using HeliQuest (http://heliquest.ipmc.cnrs.fr/). Sequence of the AH is depicted below. (B) Helical wheel representation of the p14 AH, color coded as in Fig 7. (C, D) Fusogenicity of wt p14 and p14 containing the ArfGAP1 ALPS motif in place of the p14 AH (C), and wt p15 and p15 containing the p14 AH in place of the p15 HP (D). Results are mean number of syncytial nuclei per microscopic field SD for triplicate samples from a representative experiment (n = 2).

We were interested in determining whether other FAST proteins were similarly reliant on a cytosolic lipid packing sensor. The HP of the p14 FAST protein resides in the ectodomain, not the cytosolic endodomain, where it functions as a fusion peptide to promote early stages of membrane fusion [36]. However, we noted the presence of a previously unrecognized 22-residue AH (p14 residues 70–92; sequence—LTEFQKRYLRNSYR LSEIGRPIS; hydrophobic moment—0.402) located membrane-proximal and immediately downstream of the polybasic motif in the p14 endodomain (Fig 9A and 9B). A p14 construct containing an ArfGAP1 ALPS motif substitution of this AH retained ~60% cell-cell fusion activity relative to wt p14 (Fig 9C), while p15 containing the p14AH in place of the p15 HP retained ~40% fusion activity (Fig 9D). These results suggested the FAST proteins may be generally reliant on a cytosolic, membrane-proximal curvature sensor for cell-cell fusion.

## Discussion

Formation and stabilization of the highly curved rim of a fusion pore is the rate limiting and most energy-dependent event in membrane fusion [26–28, 56]. How viral membrane fusion proteins overcome this barrier is unclear. We now show that a membrane-proximal hydrophobic patch present in the cytoplasmic tail of the p15 FAST protein preferentially partitions into membranes with high positive curvature. This partitioning is based on the presence of hydrophobic defects present in highly curved membranes, and requires the p15 HP to assume a H-L-H conformation. Furthermore, the p15 HP can be functionally replaced by ALPS motifs from heterologous proteins, but not by other membrane-interactive AHs. These results identify the p15 HP as a fusion-inducing lipid packing sensor (FLiPS), and they extend the known roles of membrane curvature-sensing motifs to a novel role in cell-cell pore formation and membrane fusion. Furthermore, a previously unrecognized membrane-proximal AH in the p14 FAST protein cytoplasmic tail can functionally replace the p15 HP, and can itself be functionally replaced by a heterologous ALPS motif, implying it serves a similar curvature sensing function as the p15 HP. Other FAST proteins, including Broome reovirus p13 [9] and aquareovirus p22 [7], also contain cytoplasmic, membrane-proximal HPs, suggesting FLiPSs may be a common mechanism employed by the FAST proteins to induce pore formation.

Several lines of evidence support the role of the p15 HP as the first example of a novel type of lipid packing sensor dependent on a H-L-H conformation: (1) circular dichroism studies indicate the wt p15HPpep peptide is only partially helical in a membrane environment (Fig 2A); (2) NMR analysis indicates p15HPpep in DPC micelles comprises two small, amphipathic helices connected by a highly flexible loop (Fig 3); (3) p15HPpep peptides containing mutations of key Gly and Pro residues in the loop yield CD spectra indicative of increased helicity, consistent with secondary structure predictions (Fig 2A); (4) these same HP mutations in the context of the p15 protein abrogate syncytium formation, inhibiting events occurring at, or upstream, of cell-cell pore formation (Fig 1); (5) the wt p15HPpep, but not mutant versions of this peptide, preferentially partitions into small liposomes with increased membrane curvature (Figs 4 and 6), and this partitioning is blocked by masking hydrophobic defects using bis-ANS (Fig 5). Thus, curvature sensing by the p15HPpep peptide and cell-cell fusion mediated by the p15 protein both directly correlate with a H-L-H architecture in the p15 HP.

**Fig 10:**
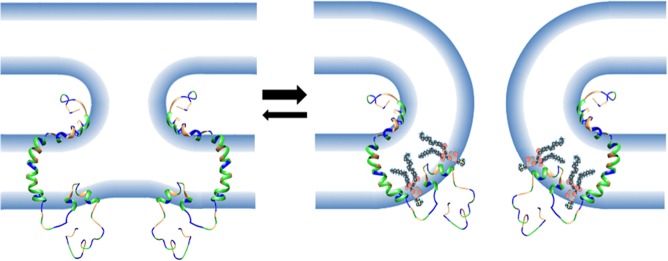
Model of the role of the FLiPS motif in membrane fusion. Depicted is a FAST protein structure based on composite NMR structures of p14 ectodomain and transmembrane domain conformers, and a p15 endodomain FLiPS conformer. Linker regions and the extended C-terminal tail (which is intrinsically disordered) are modelled as unstructured loops. Residues are colour-coded: green, hydrophobic; blue, polar/charged; orange, neutral. Fusion peptide motifs in FAST protein ectodomains are predicted to combine with essential residues in the external interfacial region of the transmembrane domain [34] to alter membrane curvature leading to dimple formation and merger of outer bilayer leaflets creating a presumed hemifusion intermediate (left). Negative curvature stresses in the outer monolayer create corresponding positive curvature stresses in the inner monolayer, rendering this transient intermediate energetically unfavorable and likely to revert to two planar bilayers. However, FLiPS partitioning into regions of increasing positive curvature, such as those in the inner monolayer at the base of the stalk-like hemifusion intermediate, would stabilize curvature stresses and promote further positive curvature making the forward reaction to pore formation (right) a more energetically favorable means to resolve the unstable hemifusion intermediate curvature. POPC molecules show insertion of the FLiPS into a hydrophobic defect generated by curvature stresses forcing apart lipid headgroups.

The small size of the FAST protein ectodomains indicates their mechanism of mediating membrane fusion cannot rely on membrane stresses induced by complex refolding of large ectodomains, as occurs with enveloped virus fusion proteins [10]. The newly defined curvature-sensing properties of the p15 HP described in this report provide a means for FAST proteins to overcome curvature stresses and lower the energy barrier to pore formation (Fig 10). During the fusion reaction, curvature changes in the outer membrane leaflet are needed to initiate merger with the outer monolayer of a closely apposed target cell membrane. In the case of FAST proteins, these curvature changes are mediated by a fusion peptide motif in the ectodomain and by essential residues in the outer interfacial region of the transmembrane domain [34–36, 38]. The presumed stalk-like hemifusion intermediate that arises from these lamellar rearrangements [57] has strong negative curvature in the outer monolayer (i.e., lipid headgroups are forced together and lipid tails splay apart). Coupling of the bilayer leaflets means the inner monolayer has to assume increasing positive curvature at the base of the stalk structure (i.e., lipid headgroups are forced apart). These curvature stresses render the hemifusion intermediate a thermodynamically unstable structure, which must either revert to two planar bilayers or advance to pore formation to resolve curvature stresses. However, pore formation requires induction of further positive curvature to merge the inner monolayers, creating an energetic barrier to pore formation and making the reverse reaction a more likely outcome [58]. Anchored near the cytoplasmic side of the plasma membrane by the p15 transmembrane domain, the p15 HP is perfectly positioned to function as a fusion-inducing lipid packing sensor (FLiPS). By partitioning into hydrophobic defects near the base of an emerging hemifusion structure to stabilize positive curvature, the FLiPS would promote stalk formation and allow further positive curvature to develop resulting in a seamless transition to pore formation (Fig 10). Hence, the FLiPS may serve as both a curvature sensor and curvature inducer. This tightly coupled hemifusion-pore formation model may explain the difficulty in detecting a hemifusion intermediate during FAST protein-mediated membrane fusion [59].

A surprising result was the ability of heterologous ALPS motifs to functionally replace the p15 HP, and this phenotype was ablated by mutations that disrupt the curvature sensing function of the ALPS motifs [47, 51, 52] (Fig 7). These observations imply linear, amphipathic ALPS motifs and the H-L-H p15 HP impart a similar curvature sensing ability to the p15 endodomain. How might these two disparate structures provide a similar function during the fusion reaction? ALPS motifs contain one or more bulky aromatic residues on their hydrophobic face and have considerably higher hydrophobic moments than the p15 HP (Fig 7), properties well-suited for interaction with curved membranes and hydrophobic defects [60]. The hydrophobic moment of AHs, while required, is not the sole determinant of curvature sensing since other AHs such as those of HO-NBAR and DivIVA have similar hydrophobic moments as the ALPS motifs but do not sense or induce positive curvature [53, 55]. Presumably, other features of these AHs influence how they interact with and insert into membranes. Unlike ALPS motifs, the p15 HP is devoid of aromatic residues and comprises predominantly hydrophobic or apolar residues resulting in limited amphipathic character when modeled as a straight helix (Fig 7). However, the two small terminal helices in the H-L-H conformation are amphipathic; residues L82, L83, V85, I86 are on one face of the C-terminal helix, and residues L70, L71 and possibly L68 in the N-terminal helix present another hydrophobic face (Fig 3). Connected by a highly flexible loop region, these two helices are free to change their relative orientation to each other; the structure of some conformers actually resembled helical hairpins, similar to conformations of the caveolin intramembrane domain [61] and the influenza virus HA fusion peptide [62]. The dynamic H-L-H structure may therefore be required to reposition the helical hydrophobic faces in the correct geometry to allow the p15 HP to optimally interact with, and insert to the correct depth into, hydrophobic defects.

The dependence of the p15 HP on a dynamic H-L-H conformation for membrane fusion activity is consistent with the phenotype of mutant p15 proteins and p15HPpep peptides. Mutations in the loop region that increased the helicity of the p15HPpep peptides resulted in loss of curvature sensing capability. Their increased retention times on reverse-phase HPLC columns (Fig 6 vs Fig 4) indicate these mutant peptides are also considerably more hydrophobic than wt p15HPpep, which presumably explains their increased association with liposomes independent of liposome size and curvature (Fig 6). In the context of the p15 protein, deeper insertion of these hydrophobic helices into the cytoplasmic leaflet would force apart lipid acyl chains and induce negative curvature, the opposite curvature needed for pore, which may explain why these HP mutations abrogate p15-induced cell-cell pore formation (Fig 1).

Membrane-proximal AHs or hydrophobic regions have also been noted in the cytoplasmic tails of many enveloped viral fusogens from diverse viruses, including retroviruses, lentiviruses, rhabdoviruses, herpesviruses and paramyxoviruses [63]. The functional significance of these AHs is not well established since tail truncations of several of these proteins retain pore forming capability [64–66]. In the case of MoMuLV, differential scanning calorimetry indicates the env protein AH induces positive curvature in membranes [67], suggesting this motif may induce or stabilize positive curvature to promote pore formation, similar to the p15 FLiPS. However, a tail truncation that deletes the MoMuLV AH has no effect on fusion pore formation, but it does partially inhibit syncytia formation [63, 66]. The MoMuLV AH may therefore promote pore expansion, not pore formation. A similar role for the cytoplasmic tails of the paramyvovirus and influenza virus fusion proteins in pore expansion has also been reported [68, 69]. Cellular curvature-generating proteins such as dynamin, ENTH domain proteins, and BAR domain proteins have also recently been shown to promote pore expansion during cell-cell fusion and vesicle exocytosis [70, 71], and the p15 HP may serve a similar role in stabilizing and propagating positive curvature in the rim of laterally expanding fusion pores. Positive curvature sensors are therefore involved in pore expansion, and as we now show for the first time, in pore formation.

## Materials and Methods

### Cells and antibodies

Vero cells were obtained from the American Type Culture Collection (ATCC) and QM5 cells were obtained from Charles Ordahl [72] and were cultured as previously described [4]. The production of rabbit polyclonal p14- and p15-specific antisera has been previously described [5, 73]. Monoclonal mouse anti-FLAG or anti-actin (Sigma-Aldrich), and Alexa Fluor 647- or horseradish peroxidase-conjugated goat-anti rabbit and goat anti-mouse conjugated secondary antibodies (Jackson ImmunoResearch) were from the indicated suppliers.

### Cloning

All FAST proteins were cloned into pcDNA3 mammalian expression vector (Invitrogen) as previously described (Corcoran and Duncan, 2004; Dawe et al., 2005). The Stratagene QuikChange mutagenesis method was used to generate alanine scan mutants, using pcDNA3-p15 as a template. A triple FLAG tag was added to the N-terminus of the indicated p15 constructs by PCR amplification for use in cell-surface immunofluorescence. The pcDNA3-p15 plasmid was used as a template to perform PCR-driven overlap extension [74] to replace the hydrophobic patch with heterologous amphipathic helices. Custom oligonucleotide primers were purchased from IDT, and all constructs were confirmed by sequencing.

### Transfection and syncytial indexing

Cells at ~70% confluency were used for transient transfection with polyethylenimine (Polysciences Inc.) 24 h after seeding. Cell monolayers were fixed with methanol at various timepoints and stained with Wright-Giemsa stain (Siemen’s Healthcare). Images were captured on a Nikon Diaphot TMD inverted microscope using the 20x objective. Fusogenicity of constructs was determined by counting the number of syncytial nuclei per microscopic field as previously described (Corcoran et al, 2004). Results are reported as the mean number of syncytial nuclei per field of view.

### Pore formation assay

Pore formation was quantified as previously described [32]. Briefly, sparsely seeded QM5 cells were co-transfected with pEGFP and the p15 construct of interest and incubated for 9 h post-transfection. Vero target cells were loaded with CellTrace calcein red-orange AM (Molecular Probes) and incubated in growth media for 2–4 h. Target cells were then resuspended with trypsin and overlaid on the transfected donor cells. At 3 h after co-culturing, cells were resuspended into PBS by trypsin digestion and fixed in suspension at 4°C using 3.7% formaldehyde. Fixed cells were analyzed by flow cytometry (FACSCaliber, Becton Dickinson), quantifying the extent of calcein red fluorescence in 10,000 gated EGFP-positive cells, indicative of cytoplasmic transfer and pore formation between donor and target cells, using FCS Express 2.0 (DeNovo software). Donor cells transfected with pEGFP plus empty vector served as negative controls.

### Cell surface expression

Transfected QM5 cells expressing N-terminally FLAG-tagged p15 constructs were incubated at 4˚C for 30 min in blocking buffer (5% normal goat serum, 1% bovine serum albumin (BSA), 0.02% NaN_3_ in HBSS) and stained with a 1:1000 dilution of anti-FLAG antibody and a 1:2,000 dilution of Alexa 647-conjugated goat anti-mouse secondary antibody, each for 1 h at 4˚C. Cells were resuspended in phosphate buffered saline (PBS) containing 10 mM EDTA, fixed with 3.7% formaldehyde, and 10,000 cells were analyzed by flow cytometry (Becton Dickinson FACSCalibur) using De-Novo software. The fluorescence gate was set to <5% for negative control cells transfected with empty vector, and the same gate was applied to quantify surface fluorescence of the p15 transfected cells. Background fluorescence from vector-transfected cells was subtracted before calculating mean percent surface fluorescence.

### Peptides

Wild-type and mutant p15HPpep peptides were synthesized by GenScript using click peptide synthesis [75] and purified to >95% purity by reverse-phase HPLC. Sequences are as follows: wt p15HPpep, acetyl-LGLLSYGAGVASLPLLNVIA-amide; p15HPpep GAG, acetyl-LGLLSY**A**A**A**VASLPLLNVIA-amide; p15HPpep G74A, acetyl-LGLLSY**A**AGVASLPLLNVIA-amide. Peptides were dissolved in DMSO (10μg/μl) at room temperature and then diluted 4-fold with DMSO/500 mM Hepes buffer, pH 8.4 (70:30, v:v) and incubated at 37°C overnight to quantitatively convert the O-acyl isopeptide bond to an N-acyl peptide bond [75]. For CD experiments, the native peptides were further concentrated using preparative reverse-phase HPLC and lyophilisation.

### Circular dichroism spectroscopy

Far-ultraviolet CD spectra of wt and mutant p15HPpep (200 μM) dissolved in 76 mM 1-palmitoyl-2-hydroxy-sn-glycero-3-[phospho-rac-(1-glycerol)] (LPPG) micelles (2:1 micell:peptide ratio) were obtained using a Jasco J-810 spectropolarimeter with an optical path of 0.1 mm. Spectra were acquired from 260 to 185 nm in 0.1 nm steps at a temperature of 37C. Measurements were collected from three scans in each of two independent experiments. Data were converted to the mean residue ellipticity, [θ], averaged, and blank.

### Liposome flotation assay

Liposomes were prepared in HEPES-buffered saline (HBS; 10mM HEPES, 150mM NaCl, pH 7.4) as previously described [36], using a 1:1:1 molar ratio of 1,2-dioleoyl-*sn*-glycero-3-phosphocholine (DOPC), 1,2-dioleoyl-*sn*-glycero-3-phosphoethanolamine (DOPE), and cholesterol (Avanti Polar Lipids) at a final total lipid concentration of 25.2 mM, and the lipid mixture was sequentially extruded through 400 nm, 100 nm and 50 nm polycarbonate filters. Electron microscopy confirmed a fairly uniform, normal distribution of liposome sizes around the mean liposome diameter expected from the filter pore size [42]. Peptides and liposomes were mixed at 1:500 peptide:lipid molar ratio (15.8 μg of peptide [75.6 nmoles] and 3.78 μmoles of lipid in a final volume of 150 μl) and incubated for 30 minutes at room temperature. The suspension was adjusted to 30% (w/v) sucrose by adding 100 L of a 75% (w/v) sucrose solution in HBS. The resulting high-sucrose suspension was overlaid with 200 μL of 25% (w/v) sucrose in HBS and 200 L of sucrose-free HBS. The sample was centrifuged at 55,000 rpm in a S55S swinging bucket rotor (206,000 x g) for 3 h at 4°C using a Sorval MTX150 benchtop ultracentrifuge. The bottom (250 μL), middle (200 μl) and top (200 μL) fractions were harvested from the bottom using a Hamilton syringe and 100 μl of the top and bottom fractions were analyzed by reverse-phase HPLC. The percent total peptide in the liposome fraction was determined by integrating the area under the HPLC peptide peaks and normalizing to the relative sample volumes.

### HPLC

HPLC was performed on a Waters 2690 Separation Module with a Vydac 214MS C4 reverse phase column (5 μm particle, 250mm × 4.6mm; Grace Davison Discovery Sciences) using a water + 0.1% trifluoroacetic acid (TFA) (Solvent A) and acetonitrile + 0.085% acetonitrile (Solvent B) mobile phase gradient (% Solvent A/B, time: [80/20, 0], [40/60, 20], [20/80, 25]) with a flow rate of 1mL/min. Peptide elution was monitored at 215nm with a Waters 2487 dual wavelength detection unit.

### Bis-ANS competition assay

Binding of 4,4′-dianilino-1,1′-binaphthyl-5,5′-disulfonic acid dipotassium salt (bis-ANS; Invitrogen) to liposomes was monitored by measuring the fluorescence after addition of a 10 mM stock solution (0.1 to 15 M final concentration) to 100 M liposomes. Fluorescence was monitored on a Varian Cary Eclipse fluorescence spectrophotometer with excitation and emission wavelengths of 395 nm and 500 nm, and a slit width of 5 nm. Liposomes were blocked with 10 M bis-ANS, pelleted by ultracentrifugation and resuspended in buffer for use in peptide-binding competition assays. Blocked liposomes were incubated with peptide under conditions identical to the liposome flotation assay described above.

### Solution NMR data acquisition and structure calculation

Atomic resolution structure of the p15HPpep (LGLLSYGAGVASLPLLNVIA; 1 mM), representing residues 68–87 of p15 FAST protein, was determined in the presence of dodecylphosphocholine (DPC-d_38_; 150 mM) micelles in 20 mM sodium acetate, 90% H2O/10% D2O at pH 5.0. 1D ^1^H, 2D ^15^N-^1^H HSQC (natural abundance), 2D ^13^C-^1^H HSQC (natural abundance), 2D ^1^H-^1^H TOCSY (80 ms mixing time), and 2D ^1^H-^1^H NOESY (200–400 ms mixing times) experiments were acquired at 37°C using a Bruker Avance III 700 MHz spectrometer equipped with a 5 mm TCI cryoprobe. Following NOE buildup analysis, distance restraints were determined to be unaffected by spin diffusion in the NOESY at 200 ms mixing time. The NOE restraints were therefore produced from NOESY cross-peak analysis at 200 ms. Following iterative refinement [46], 511 nonredundant NOEs were retained, 173 of which corresponded to intraresidue contacts, 294 to interresidue contacts, and 44 to ambiguous contacts (see S1 Table). Structure calculations were performed using the simulated annealing algorithm within the python scripting interface of XPLOR-NIH 2.34 [76] incorporating the RAMA torsion angle database potential term [77]. The 50 p15HPpep lowest energy structures out of 100 calculated were retained for analysis. Atomic coordinates are deposited in the Research Collaboratory for Structural Bioinformatics (www.rcsb.org), with PDB ID 2MNS. Chemical shifts are deposited in the Biological Magnetic Resonance Bank (www.bmrb.wisc.edu) with entry ID 19902.

## Supporting Information

S1 Table NMR statistics for the structural ensemble of p15HPpep peptide. ^1^H-^1^H pairwise distance restraints generated from observed Nuclear Overhauser Effects (NOEs), average energies, restraint violations, average pairwise root mean square deviations (RMSDs) and Ramachandran plot statistics of the final structural ensemble of the 50 lowest-energy structures.
(DOCX)

S1 Fig Substitution of essential glycine and proline residues in the p15 HP kink does not affect p15 cell surface expression. Cell surface fluorescence of QM5 cells expressing wt p15 (p15) or p15 mutant proteins containing Ala substitutions of the indicated HP residues was quantified by flow cytometry using anti-p15 antiserum and Alexa Fluor 647-conjugated secondary antibody. Results are mean fluorescence intensity ± SEM relative to wt p15 for triplicate samples from n = 3 experiments. Significance assessed by ANOVA with Tukey post-test (ns–not significant).
(TIF)

S2 Fig Solution-NMR spectra and atomic resolution structure of wt p15HPpep in DPC micelles at pH 5 and 37°C. (A) Intraresidue/interresidue ^1^H-^1^H correlations in the 2D TOCSY (red) and 2D NOESY (blue) spectra. Only a portion of the full spectra is shown. All the peaks displayed were assigned, but for clarity, not all have been labeled. (B) ^1^H-^15^N correlations in the 2D HSQC spectrum. Despite the presence of ^15^N nuclei only at a natural abundance, strong peaks corresponding to all 20 amino acids of the peptide were seen. (C) ^1^H-^13^C correlations in the 2D HSQC spectrum. Despite the presence of ^13^C nuclei only at a natural abundance, peaks corresponding to all α and β sites of the peptide were assignable. (D) Secondary chemical shift (Δδ) values for C^α^ and H^α^ resonances, calculated by subtracting the random coil chemical shifts for peptides of sequence GGXAGG measured in dimethyl sulfoxide from that of the p15_ALPS_ amino acids. Simultaneous observation of positive Δδ(C^α^) and negative Δδ(H^α^) secondary chemical shift values are consistent with an α-helical conformation at the C-terminus of the peptide.
(TIF)

S3 Fig Controls for quantitative recovery of liposomes from the top layer of sucrose gradients independent of liposome size, and linear detection range of p15HPpep by HPLC. (A) Liposomes were labeled with 1% 1,2-dioleoyl-sn-glycero-3-phosphoethanolamine-N-(7-nitro-2-1,3-benzoxidiazol-4yl) (NBD) and extruded to varying diameters (400 nm, 100 nm, or 50 nm). Liposomes were loaded on the bottom of a centrifuge tube, adjusted to 30% (w/v) sucrose and overlaid with 25% (w/v) sucrose and a top buffer layer. Gradients were centrifuged at 240,000 *x g* for 1h to float liposomes. The layers of the gradient were harvested and solubilized with 1.5% SDS and NBD fluorescence was determined. All fluorescent readings fell within a linear detection range indicating equivalent quantitative recovery of different sized liposomes from the sucrose gradient. % Fluorescence = (F_Top_ / F_Input_). F_Input_ was determined by reading fluorescence of liposomes prior to loading on gradient. (B) Linear detection range of wt p15HPpep by HPLC. Eluted peptide was detected at 215 nm and the corresponding area under the peak was quantified using Waters software.
(TIF)
