# Supplementary material for: Reovirus FAST Proteins Drive Pore Formation and Syncytiogenesis Using a Novel Helix-Loop-Helix Fusion-Inducing Lipid Packing Sensor
Source: PLoS Pathog. 2015 Jun 10;11(6):e1004962. doi: 10.1371/journal.ppat.1004962 (PMC4464655; doi:10.1371/journal.ppat.1004962)
Supplement: S1 Table — 1H-1H pairwise distance restraints generated from observed Nuclear Overhauser Effects (NOEs), average energies, restraint violations, average pairwise root mean square deviations (RMSDs) and Ramachandran plot statistics of the final structural ensemble of the 50 lowest-energy structures. (DOCX) [file ppat.1004962.s001.docx]

**Supplementary Table 1:** NMR statistics for the 50-member structural ensemble of p15HPpep peptide.

Distance Restraints

Unique NOEs : 511

Intra-residue : 173

Sequential (i and i ± 1) : 173

Medium-range (i and i ± 2, 3, 4) : 117

Long-range (i and i ± 5) : 4

Ambiguous : 44

XPLOR-NIH average energies with standard deviations (kcal/mol)

Total : –126.87 ± 15.60

NOE : 2.28 ± 1.43

RAMA : –165.40 ± 15.28

Restraint violations in any of the structures

NOE violations > 0.1 Å : 0

Average pairwise RMSDs (Å) (8)

Backbone atoms (all residues) : 3.35 ± 0.94

Backbone atoms (residues L71-G74) : 0.59 ± 0.48

Backbone atoms (residues P81-I86) : 0.12 ± 0.04

Ramachandran plot statistics (%) (7)

Most favored : 78.6

Additional allowed : 15.0

Generously allowed : 6.0

Disallowed : 0.4
