# Supplementary figures and images for: Reovirus FAST Proteins Drive Pore Formation and Syncytiogenesis Using a Novel Helix-Loop-Helix Fusion-Inducing Lipid Packing Sensor
Source: PLoS Pathog. 2015 Jun 10;11(6):e1004962. doi: 10.1371/journal.ppat.1004962 (PMC4464655; doi:10.1371/journal.ppat.1004962)

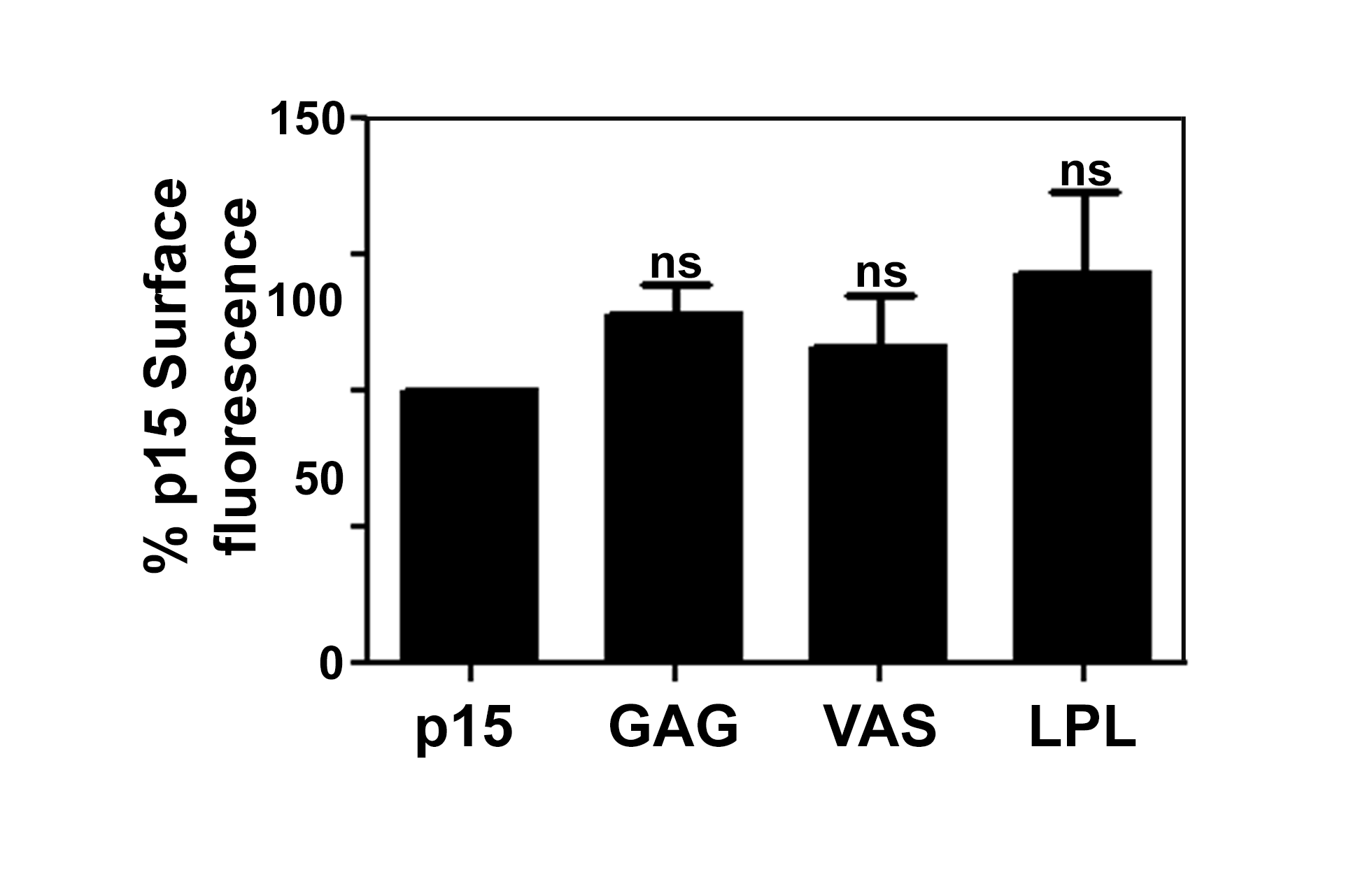

Supplement: S1 Fig — Cell surface fluorescence of QM5 cells expressing wt p15 (p15) or p15 mutant proteins containing Ala substitutions of the indicated HP residues was quantified by flow cytometry using anti-p15 antiserum and Alexa Fluor 647-conjugated secondary antibody. Results are mean fluorescence intensity ± SEM relative to wt p15 for triplicate samples from n = 3 experiments. Significance assessed by ANOVA with Tukey post-test (ns–not significant). (TIF) [file ppat.1004962.s002.tif]

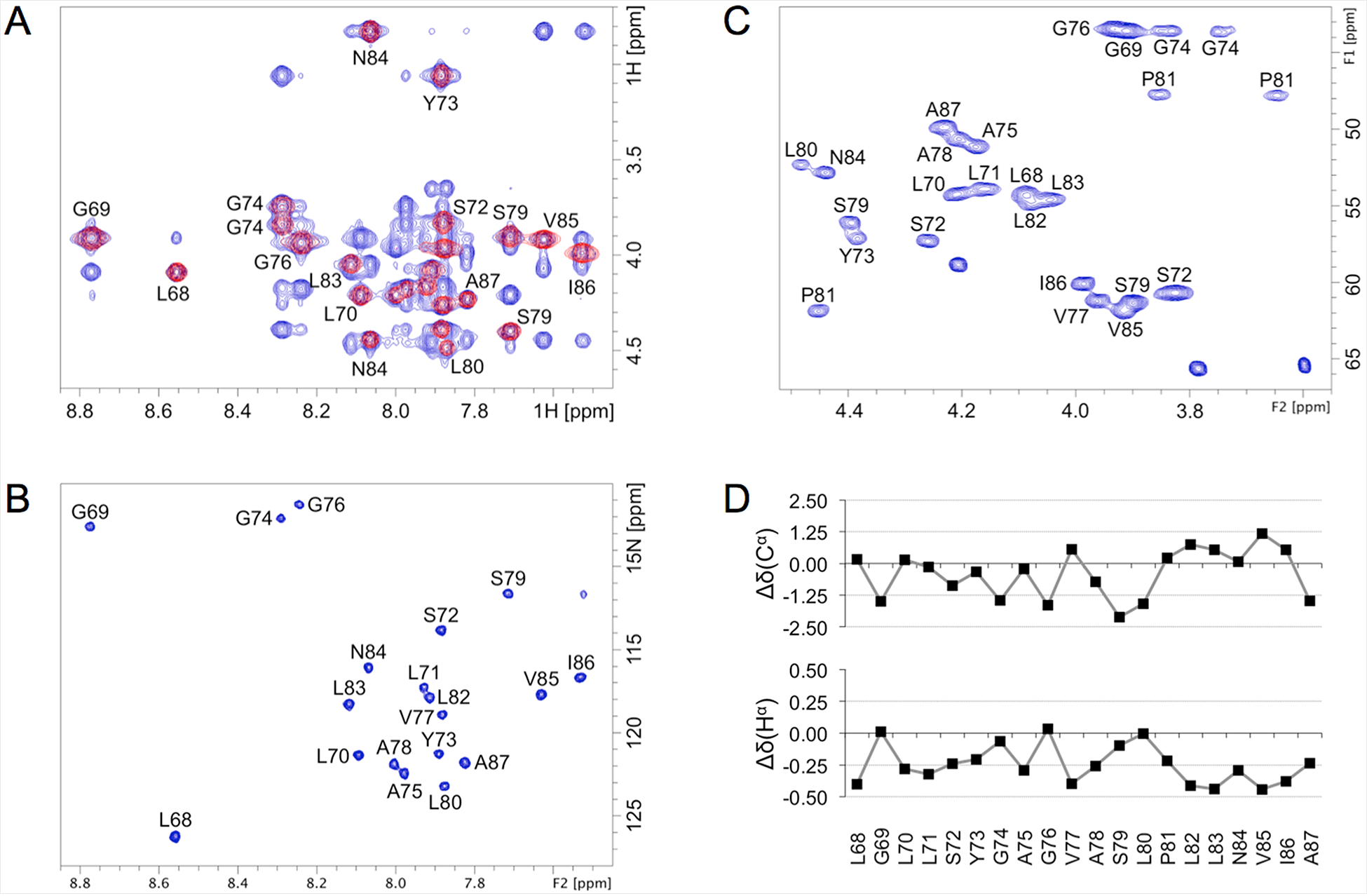

Supplement: S2 Fig — (A) Intraresidue/interresidue 1H-1H correlations in the 2D TOCSY (red) and 2D NOESY (blue) spectra. Only a portion of the full spectra is shown. All the peaks displayed were assigned, but for clarity, not all have been labeled. (B) 1H-15N correlations in the 2D HSQC spectrum. Despite the presence of 15N nuclei only at a natural abundance, strong peaks corresponding to all 20 amino acids of the peptide were seen. (C) 1H-13C correlations in the 2D HSQC spectrum. Despite the presence of 13C nuclei only at a natural abundance, peaks corresponding to all α and β sites of the peptide were assignable. (D) Secondary chemical shift (Δδ) values for Cα and Hα resonances, calculated by subtracting the random coil chemical shifts for peptides of sequence GGXAGG measured in dimethyl sulfoxide from that of the p15ALPS amino acids. Simultaneous observation of positive Δδ(Cα) and negative Δδ(Hα) secondary chemical shift values are consistent with an α-helical conformation at the C-terminus of the peptide. (TIF) [file ppat.1004962.s003.tif]
